# A Sensitivity-Enhanced Sub-Zero Temperature Sensor Based on the Mach–Zehnder Interferometer Coated with Organic Silicone Film

**DOI:** 10.3390/mi17070792

**Published:** 2026-06-28

**Authors:** Yibo Zhang, Shunxing Wang, Haoran Xu, Siyang Yu, Cuiting Sun

**Affiliations:** College of Science, Northeast Forestry University, Harbin 150001, China; zhangyibo@nefu.edu.cn (Y.Z.); 2023213222@nefu.edu.cn (S.W.); 2023213226@nefu.edu.cn (H.X.); 2024213033@nefu.edu.cn (S.Y.)

**Keywords:** sub-zero temperature measurement, Mach–Zehnder interferometer, sensitivity-enhanced sensor

## Abstract

In this paper, a high-sensitivity sub-zero temperature sensor based on the Mach–Zehnder interferometer coated with organic silicone film is proposed. The sensor is fabricated by polishing four orthogonal sides in the multimode fiber (MMF) of the single-mode–multimode–single-mode (SMS) structure with a CO_2_ laser and then coating the structure with a mixed film of hydroxyl-terminated siloxane and methyl MQ silicone resin to form a sub-zero-sensitive layer. The sub-zero temperature sensitivity of the coated sensors is experimentally compared for samples with different numbers of polished facets. The experimental results show that the sub-zero temperature sensitivity of the sensors with one to four polished facets increased from 0.73 nm/K to 2.15 nm/K in the temperature range of 223 K to 273 K. The fiber sub-zero sensor has the advantages of compact structure, high sensitivity and low cost, which lead to the potential for application in fields such as biomedicine, aerospace and energy.

## 1. Introduction

Sub-zero temperature measurement is required in a wide range of applications, including aerospace systems, superconducting facilities, energy storage and transportation, and biomedical environments, where reliable thermal monitoring is directly related to safety and performance. Conventional sub-zero temperature detection is still mainly realized with electrical sensors such as thermocouples, resistance thermometers and semiconductor devices [1]. However, in harsh or space-limited environments, these sensors are often constrained by electromagnetic interference, wiring complexity and thermal loading effects. Optical fiber sensors are well suited to this task because of their compact size, corrosion resistance, immunity to electromagnetic interference and multiplexing capability [2].

A variety of fiber-optic configurations have been explored for sub-zero temperature sensing, including Sagnac interferometers (SIs) [3], long-period fiber gratings (LPFGs) [4], Fabry–Perot interferometers (FPIs) [5], and Mach–Zehnder interferometers (MZIs) [6]. For example, Cai et al. proposed an SI based on the Vernier effect in a polarization-maintaining fiber, which achieved a sub-zero sensitivity of 1.74 nm/K [7]. However, SIs have inherent drawbacks such as large size and low robustness, which make them unsuitable for device miniaturization. In addition, the Vernier effect also poses a challenge to the demodulation of the sensor. In contrast, MZIs have advantages such as excellent design flexibility, simple deposition of a sensitive layer, and low fabrication cost. These features make them highly suitable for sub-zero sensing applications. For example, an MZI based on a dual spherical single-mode–multimode–single-mode fiber structure was proposed for sub-zero measurement with a sensitivity of −0.03728 nm/K [8]. The sensors are relatively simple to prepare but have relatively low sensitivity. The methods for improving sensor performance include polishing technology and coating technology. For example, Zhang et al. proposed a side-polished fiber MZI based on a graphene oxide coating [9]. The temperature sensitivity of the sensor reaches 0.132 nm/K. However, graphene oxide is expensive and becomes inactive at low temperatures, which is unsuitable for sub-zero environments. Sampath et al. fabricated an epoxy-coated side-polished fiber sensor for sub-zero conditions [10]. This sensor demonstrated the application potential of side-polished fiber sensors with temperature-sensitive coatings in sub-zero sensing. But the sensor employs intensity demodulation, so it is not conducive to application in complex environments. Therefore, the demand remains strong for a coated fiber sensor that can operate stably and sensitively at low temperatures. With the development of materials science, composite films have shown excellent application potential. A mixed film of hydroxyl-terminated siloxane and methyl MQ silicone resin provides an excellent option which combines high sensitivity and low cost. This material maintains good thermal response activity at low temperatures, which enables the refractive index and the large deformation of the coating in the sensor to change effectively with temperature [11]. In addition, it does not become brittle at low temperatures, which helps maintain the structural integrity and long-term stability of the coating sensing region [12].

Based on the above advantages, we propose a sensitivity-enhanced sub-zero temperature sensor in this work. The sensor is fabricated by coating a sub-zero-sensitive composite film onto the polished multimode fiber (MMF) in the SMS structure. To further enhance the optical field coupling between the fiber and the film, the polishing geometry is then extended from one side to four sides. This allows the temperature-dependent perturbation introduced by the surrounding film to be transferred more efficiently to the MMF. Experimental results show that the highest sub-zero temperature sensitivity of the sensor reaches 2.15 nm/K with a temperature range of 223–273 K. With its compact size, straightforward fabrication and enhanced sensitivity, the proposed sensor is well suited for sub-zero temperature sensing in space-constrained applications.

## 2. Fabrication Method and Working Principle

**Figure 1:**
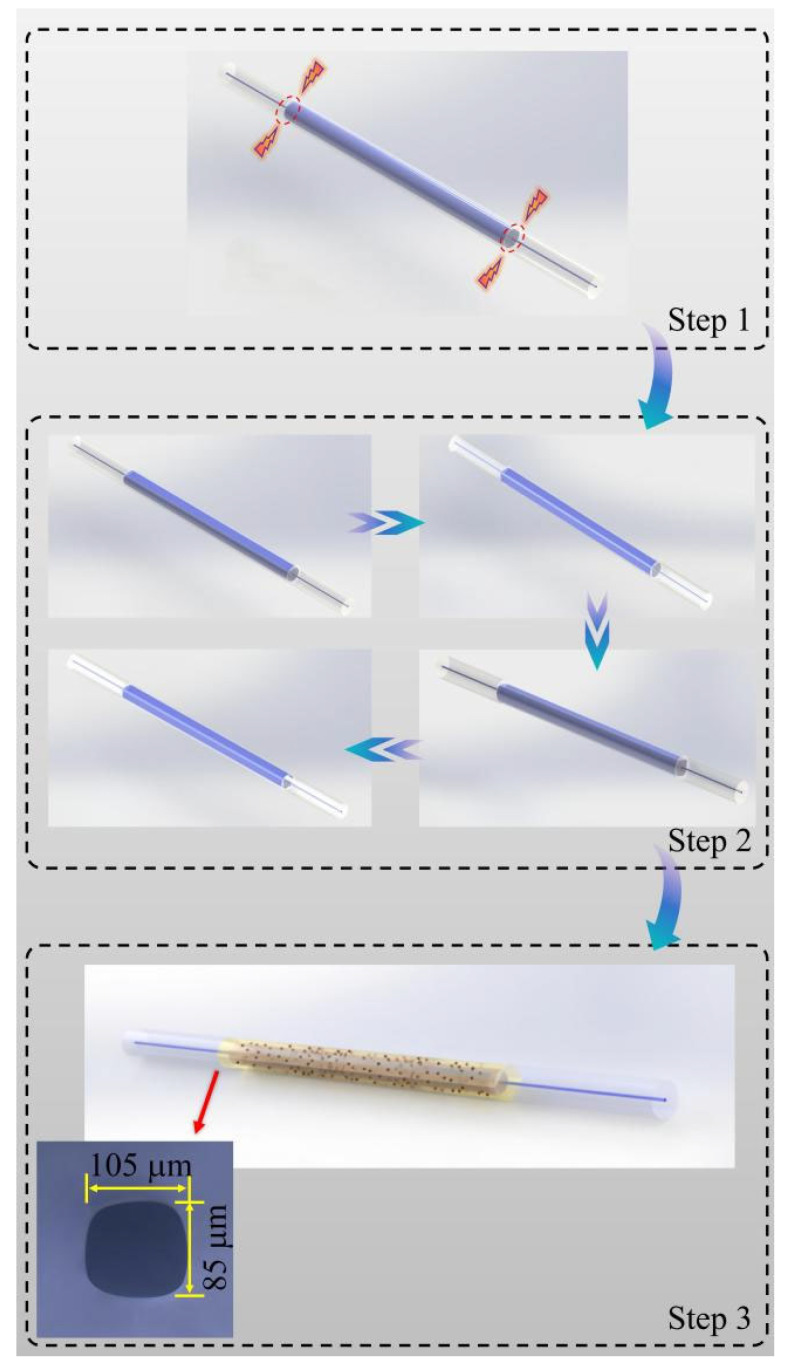
Fabrication process.

The fabrication process of the proposed sensor is described below, as shown in Figure 1. In the first step, the arc discharge technology was used to sequentially splice two single-mode fibers (SMFs, 8/125 μm) and a 2.0 mm multimode fiber (MMF, 105/125 μm), which form a single-mode–multimode–single-mode (SMS) structure. Suitable processing parameters can ensure the mechanical strength of the sensor. The experimental parameters after preliminary verification are shown in Table 1. In the second step, the prepared SMS structure was transferred to a CO_2_ laser polishing platform for laser polishing. A microscopic imaging system was used for pre-processing calibration to confirm the polishing carried out on the entire MMF. The polishing length was set as 2 mm. One side of the MMF in the SMS structure was polished using the CO_2_ laser with the parameters shown in Table 2. The processing was repeated three times. Then the structure was rotated 90° to complete the polishing of the second side. The rotation and polishing process was repeated to remove the four-layer coating of the MMF. The base structure of the sensor was completed. A microscope image of the polished MMF is illustrated in Figure 1. It can be seen that the uniformity of the four faces is good, which demonstrates good fabrication consistency.

**Figure 2:**
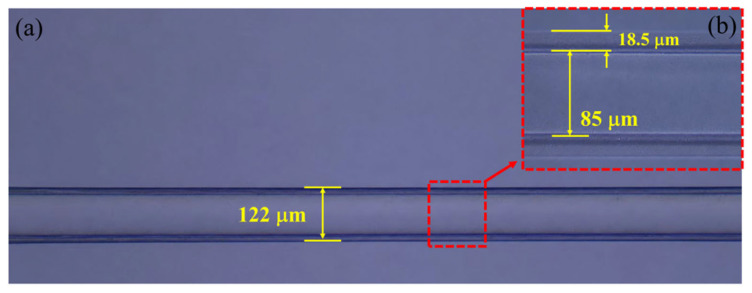
Microscope images of the coated sensor. (**a**) Overall structure. (**b**) Magnified image of the coating.

After polishing, the surface of the sensor was cleaned with isopropanol solution to remove surface impurities. In the third step of the sensor fabrication, a silicone-based temperature-sensitive coating was deposited onto the polished MMF section by the dip-coating method. The coating material was prepared by mixing hydroxyl-terminated siloxane and methyl MQ silicone resin, with benzoyl peroxide (BPO) used as the curing agent. The mass ratio of hydroxyl-terminated siloxane, methyl MQ silicone resin, and BPO was 5 to 1. After coating, the sensor was subsequently cured at 20 °C for 1 h. Microscopic images of the sensor are shown in Figure 2. It is shown that the diameter of the polished and coated fiber is 122 μm. The diameter of the polished fiber without coating is 85 μm. The coating thickness on both sides is 18.5 μm, which demonstrates good uniformity of the coating.

**Figure 3:**
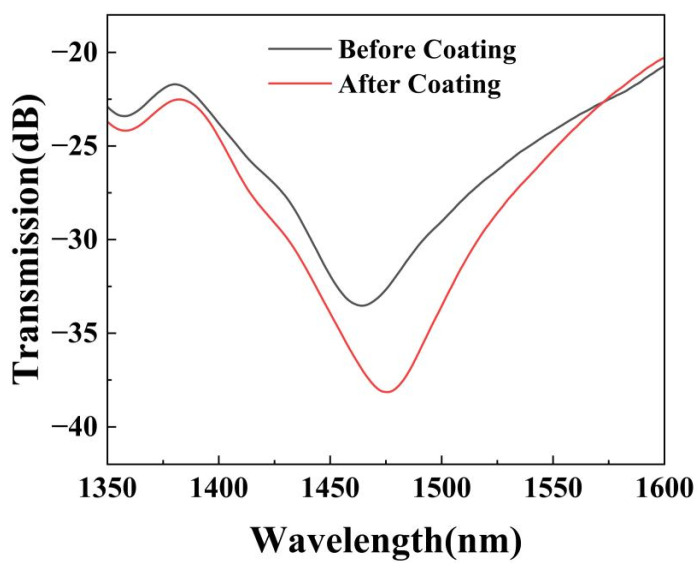
Comparison of transmission spectra of the sensor.

The transmission spectra before and after the coating of the sensor are compared in Figure 3. The results show that the coating thickness is not sufficient to support an anti-resonant effect in the structure. Therefore, the spectral changes only include a slight improvement in coupling efficiency and a shift in the resonance dip caused by the change in the refractive index. The spectral response of this sensor originates from the intermodal interference phenomenon in the MMF section. Under the condition of two-beam approximation, the output intensity can be expressed as [13]:

(1) $$I=I_{1}+I_{2}+2\sqrt{I_{1}I_{2}}\cos\left(\frac{2\pi ∆n_{eff}L}{\lambda }\right)$$

where $I_{1}$ and $I_{2}$ are the optical intensities of the two dominant interfering modes, ${\Delta n}_{eff}$ is the effective refractive-index (ERI) difference between them, and L is the effective interference length. When the phase matching condition is satisfied, the resonant wavelength of the *m*-th order mode can be expressed as follows:

(2) $$\lambda_{m}=\frac{2∆n_{eff}L}{2m+1}$$

where λ is the resonance wavelength near the considered interference dip. Accordingly, any temperature-induced variation in either L or $∆n_{eff}$ will lead to a spectral shift. Further, because the coating material has a negative thermo-optic coefficient, the thermo-optic effect increases its ERI when the temperature decreases, which leads to a blueshift of the spectrum.

**Figure 4:**
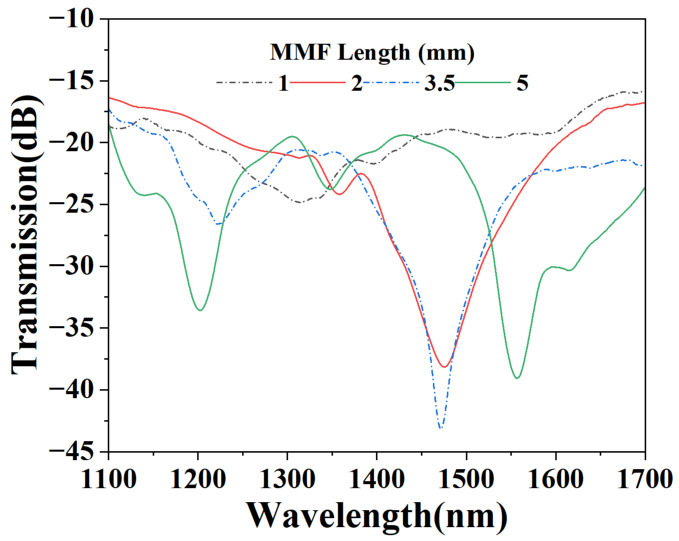
Transmission spectra of four-side-polished and coated SMS sensors with different MMF lengths.

To investigate the influence of MMF length on the modal interference spectrum, four-side-polished and coated SMS sensors with different MMF lengths were fabricated and measured under the same polishing depth. Figure 4 shows the transmission spectra of the four samples. It can be seen that the spectral characteristics strongly depend on the length of the MMF. As the length increases, the free spectral range (FSR) decreases accordingly. When the length of the MMF is too short, such as at 1 mm, the coupling between the fundamental core mode and the higher-order modes is insufficient to form a clear resonance dip. When the length of the MMF is increased to 2 mm, only one clear and isolated resonance dip appears, at 1510 nm, within the visible range of the optical spectrum analyzer (OSA, AQ6317B). This dip has an extinction ratio (ER) greater than 20 dB, which indicates good coupling at this length. When the length of the MMF is further increased to 3.5 mm and 5 mm, the number of higher-order modes that participate in the interference increases. Although increasing the MMF length can enlarge the accumulated phase difference in an ideal interferometric structure, an excessively long MMF section in the present SMS sensor excites more higher-order modes and complicates the modal beat relationship. As a result, multiple resonance dips appear in the spectrum, which introduces mode crosstalk and peak-tracking ambiguity. Therefore, the degradation predicted for the 3.5 mm and 5 mm samples is not caused by a reduced material interaction effect, but rather by the increased intermodal dispersion and reduced spectral linearity and stability. This makes the demodulation process more difficult and lowers the theoretical temperature-response performance.

## 3. Experimental Results and Discussion

**Figure 5:**
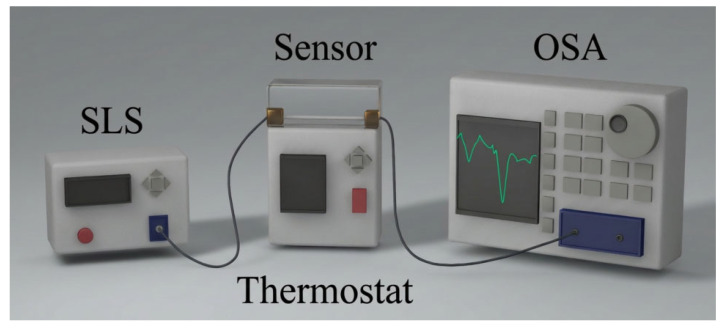
Experimental setup for sub-zero temperature characterization.

The sub-zero temperature response of the proposed sensor was characterized using the experimental setup shown in Figure 5. Broadband light from a supercontinuum light source (SLS, YSL Photonics Co., Ltd., Wuhan, China) was launched into the sensor, and an interference spectrum was generated. An OSA was used to acquire these interference spectra. During the measurement, the sensor was fixed in the temperature-controlled chamber to avoid bending-induced spectral fluctuation. The temperature was gradually varied from 223 K to 273 K, and the transmission spectrum was recorded after the temperature reached a stable state at each measurement point.

**Figure 6:**
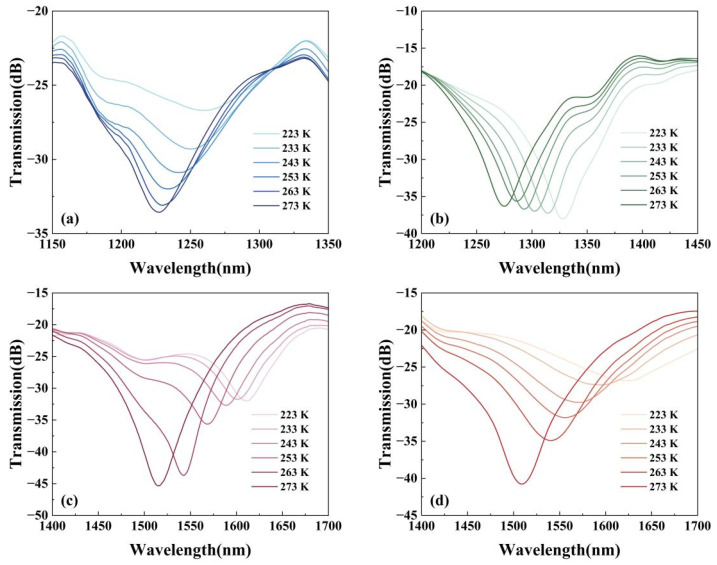
Temperature-dependent spectra of coated sensors with different polished facet numbers. (**a**) Sample 1: one facet. (**b**) Sample 2: two facets. (**c**) Sample 3: three facets. (**d**) Sample 4: four facets.

The proposed sensor was fabricated using CO_2_ laser polishing technology. For MMFs with different numbers of polished facets, the response of the sensor to temperature-induced refractive-index and shape changes of the coating material also varies accordingly. Therefore, we prepared sensor samples with different numbers of polished facets. All samples had the same polishing depth and were coated with the same thickness of the sensitive material. The spectral variations in these samples under different temperatures are shown in Figure 6. As the temperature increases, all spectra exhibit a blueshift in the wavelength. This result indicates that the coating material composed of hydroxyl-terminated siloxane and methyl MQ silicone resin has good temperature sensitivity and consistency.

**Figure 7:**
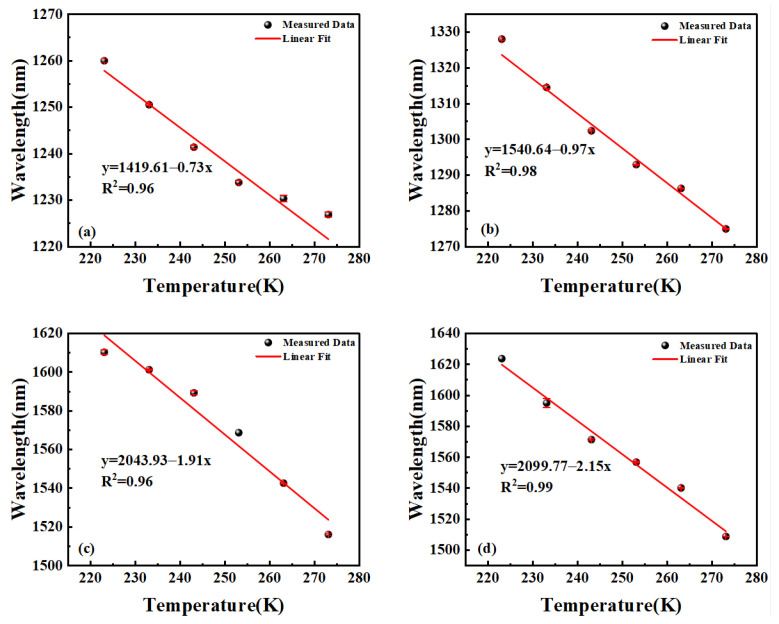
Fitted temperature sensitivity versus temperature for coated sensors with different polished facet counts. (**a**) Sample 1. (**b**) Sample 2. (**c**) Sample 3. (**d**) Sample 4.

Figure 7 shows the relationship between temperature and wavelength shift for the four samples. As the number of polished facets increases, the temperature sensitivity gradually increases. Linear fitting was used to analyze these discrete data points for the sensitivity trend and the average sensitivity. The specific values are as follows. Sample 1, with a single-side-polished MMF, has a sensitivity of −0.73 nm/K. Sample 2, with a double-side-polished MMF, has a sensitivity of −0.97 nm/K. Sample 3, with a triple-side-polished MMF, has a sensitivity of −1.91 nm/K. Sample 4, with a four-side-polished MMF, has the maximum sensitivity, achieving −2.15 nm/K. The enhancement mechanism can be understood from the perspective of evanescent field perturbation. The polished facets act as optical leakage channels that increase the overlap between guided modes and the external thermo-optic coating, which can be expressed by [14]:

(3) $$S\propto \frac{\partial \lambda }{\partial T}\propto \Gamma_{eff}\cdot \frac{\partial n_{caoting}}{\partial T}$$

where *S* denotes the sensitivity of the sensor, *Γ_eff_* represents the effective modal overlap factor, and *n_coating_* is the ERI of the coating layer. As the number of polished facets increases, the effective interaction parameter *Γ_eff_* is enhanced because additional leakage interfaces are introduced. These interfaces strengthen the interaction between the modes and the coating. Since the modal field distribution is non-uniform across the MMF cross-section, each facet contributes differently. This results in a nonlinear increase in *Γ_eff_* with the facet number. Consequently, the overall sensitivity *S* increases as the number of polished facets grows. When all four facets are polished, the interaction approaches a near-saturation state. In this state, further enhancement becomes limited. In addition, measurement errors originate from the insufficient accuracy of the temperature control equipment, which leads to small fluctuations in the spectrum. However, because the magnitude of these fluctuations is small, they can be considered negligible in practical applications.

**Figure 8:**
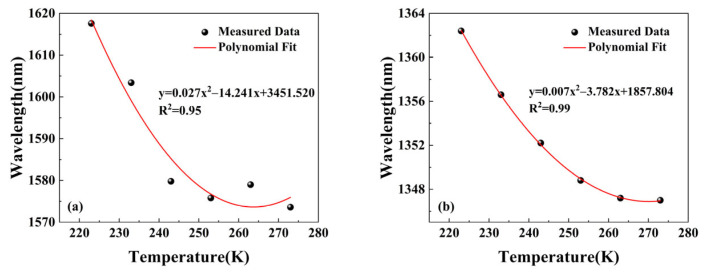
Temperature-dependent wavelength responses and quadratic fitting results for the four-side-polished coated sensors. (**a**) 3.5 mm sensor. (**b**) 5 mm sensor.

In addition, to verify whether the performance of the sensor degrades as theoretically predicted when the interference length becomes too long, temperature tests were conducted on samples with lengths of 3.5 mm and 5 mm. The wavelength responses of two sensors to temperature are shown in Figure 8. It can be calculated that the average sensitivities of the 3.5 mm and 5 mm sensors are 0.85 nm/K and 0.31 nm/K, respectively. The experimental results are consistent with the theoretical analysis. The longer the sensor is, the lower its performance will be in sub-zero temperature environments. Although these two samples have suitable extinction ratios and free spectral ranges, the nonlinearity of the test results limits their practical application. This is because an excessively long structure introduces multiple higher-order modes, and the mode beats generate higher-order Fourier coefficients. Consequently, the relationship between the ambient temperature change and the resonant wavelength of the sensor cannot be fitted with a simple expression, which increases the difficulty of demodulation. Therefore, an interfering structure with a length of 2 mm is selected to be the sensor for practical applications.

**Figure 9:**
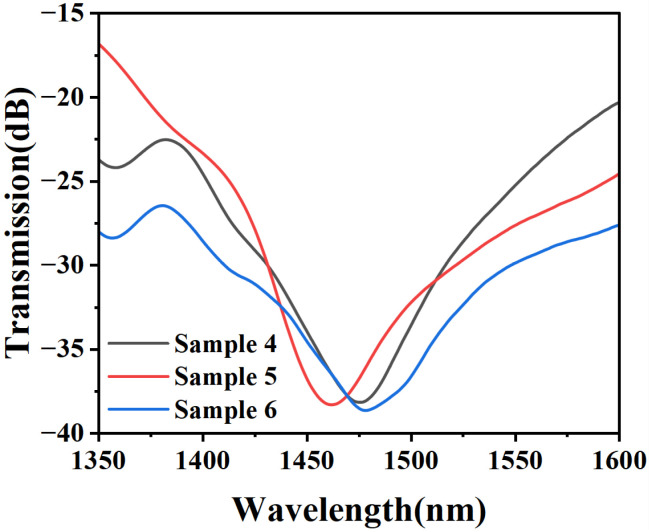
The spectra of several different samples.

**Figure 10:**
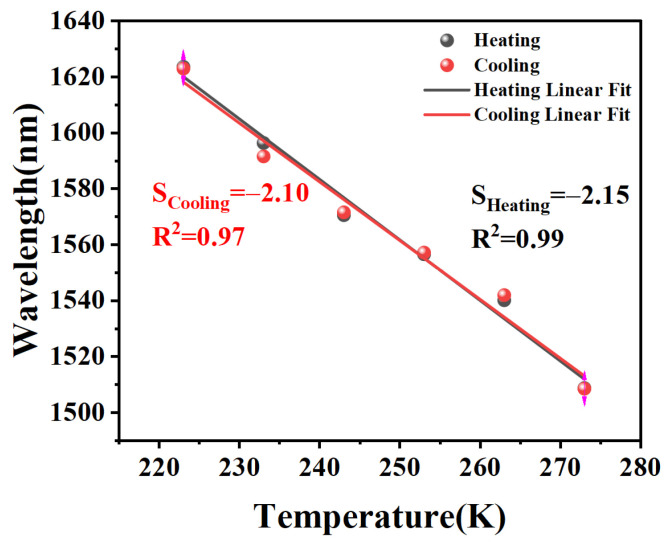
Wavelength–temperature relationship of the proposed sensor during heating and cooling.

To verify the fabrication repeatability of the proposed structure, several different samples were prepared. Their spectra are shown in Figure 9. Under the same fabrication and test conditions, the wavelengths of the three samples are almost identical. This indicates that the fabrication method is stable and that the repeatability among the samples is good. In addition, the measurement accuracy of the sensor was investigated to demonstrate the performance of the samples. The resolution of the proposed sensor is given by the following expression:

(4) $$R_{T}=\frac{\sigma_{\lambda }}{S}$$

where *σ_λ_* represents the statistical fluctuation of the center wavelength of the spectral line. This fluctuation is obtained from repeated measurements at the same temperature point. *S* is the fitted temperature sensitivity of the sensor. For the spectral data collected repeatedly at the same point, *σ_λ_* is calculated as the standard deviation of the center wavelengths from multiple measurements, which characterizes the wavelength uncertainty of the measurement system. This approach eliminates the influence of factors such as spectral line fluctuations and environmental disturbances. For the proposed sensor, the statistical fluctuation *σ_λ_* under three repeated measurements is 0.705 nm. The calculated resolution is 0.328 K. Based on these results, the performance of the sensor during heating and cooling was tested to evaluate its long-term operational stability. The test results are shown in Figure 10. Within a complete heating and cooling cycle, the sensor operated without significant performance degradation. This indicates that the sensor has excellent long-term operational stability.

After the parameters of the sensor were determined, the performance of the sensor needed to be evaluated. Table 3 compares the performance of the sensor reported in this work with those of several fiber-optic temperature sensors reported in recent years [2,3,7,8,9,15,16,17,18,19]. The proposed coated sensor demonstrates a significantly more compact structure and higher sensitivity than most of the listed sensors. Although its sensitivity is lower than that of the Vernier-effect structure [19], the length of the proposed sensor is much shorter. Under the same length condition, the sensitivity of the proposed sensor is significantly higher than those of the other sensors. This result confirms the superior performance of the sensor. Therefore, the proposed coated sensor is promising for applications in harsh low-temperature environments such as aerospace and oil and gas pipelines.

## 4. Conclusions

In this work, a four-side-polished MZI sensor with a composite coating is proposed for sub-zero temperature measurement. The sensor is fabricated by CO_2_ laser polishing and dip-coating of a hydroxyl-terminated siloxane and methyl MQ silicone resin mixture. The influence of the MMF length and the number of polished facets in the sensor on the spectral response was systematically investigated. As the number of polished facets increases, the temperature sensitivity rises progressively, reaching a maximum of 2.15 nm/K for the four-side-polished structure. Compared with recently reported fiber-optic temperature sensors, the proposed sensor exhibits small size and superior sensitivity and resolution. The proposed sensor is promising for harsh sub-zero temperature applications such as aerospace and oil–gas pipelines.

## Figures and Tables

**Table 1:** The parameters used for arc discharge.

Parameter	Value
Discharge power	125–136 bit
Discharge time	1800 ms
Discharge overlap length	10 μm

**Table 2:** The parameters used for CO_2_ laser polishing.

Parameter	Value
Scanning speed	60 mm/s
Laser power	21 W
Pulse frequency	20 kHz

**Table 3:** The performance comparison between the sensor and proposed sub-zero temperature sensors.

Ref.	Configuration	Temperature Sensitivity (nm/K)	Sensor Length (mm)
[2]	FBG	0.048	25
[3]	LPFG	0.85	20
[7]	SI	1.74	15
[8]	MZI	0.03728	9.01
[9]	MZI	0.13177	30
[15]	FBG	0.0107	5
[16]	FBG	0.029	5
[17]	MMI	0.1065	23
[18]	MI	0.0775	11
[19]	Vernier effect	56.95	>1000
This work	MZI	2.15	2

## Data Availability

The data presented in this study are available on request from the corresponding author.
